# Ten Simple Rules of Live Tweeting at Scientific Conferences

**DOI:** 10.1371/journal.pcbi.1003789

**Published:** 2014-08-21

**Authors:** Sean Ekins, Ethan O. Perlstein

**Affiliations:** 1 Collaborations in Chemistry, Fuquay-Varina, North Carolina, United States of America; 2 Collaborative Drug Discovery, Burlingame, California, United States of America; 3 Perlstein Lab, San Francisco, California, United States of America; National Institutes of Health, United States of America

The power of mobile communications has increased dramatically in recent years such that these devices (smartphone or tablet computer) can be used productively to do science [1]. The software applications installed on them do not necessarily have to be specialized to be useful for science, e.g., Evernote can be used as an electronic lab notebook [2]. Twitter is a popular microblogging platform famously limited to messages of up to 140 characters [3] and represents a simple way to express what's on your mind to a global audience of followers. Twitter has useful real-world scientific applications, such as in disease surveillance enabling the tracking of disease pandemics [4]–[6], as well as the capacity to be used for the communication of science itself [7]. Like other professionals, scientists are increasingly tweeting about their own research and the work of colleagues and sharing links to scholarly publications, laboratory results, and related scientific content such as molecular structures [8]. Twitter can additionally serve as a catalyst in the development of scientific tools, with at least one mobile app for science coming directly out of a tweet at a scientific conference [9].

If he or she is fortunate, a scientist may attend one or more scientific conferences in a year. In some fields, the number of conferences to attend is overwhelming. The time and cost expenditures required to physically participate in conferences necessitate an alternative route to access the information presented and capture it for future reference. Ideally, it would be preferable to monitor conferences remotely and at minimal or no cost. Increasingly, some scientists are using Twitter as a vehicle to summarize presentations and posters at conferences in real time, which is defined as “live tweeting.” The advantage of remote participation is that the information tweeted is open and free to anyone around the globe (Figure 1). From our own experiences of attending and live tweeting at several conferences over the past three years, the success of live tweeting appears dependent on the engagement of conference organizers with Twitter and its active encouragement before, during, and after the meeting. Surprisingly few conferences are actively encouraging scientists to tweet. This reticence is probably more likely due to ignorance of the potential rather than the possibility of loss of attendee revenue. We suggest that conference live tweeting is an opportunity to reach beyond those in the room while enabling feedback from those outside. Obviously, it is also in the best interests of conference organizers to provide free Wi-Fi so that international attendees do not have to use their expensive data plans and because the phone signal in many conference venues is generally weak. Crucially, the success of live tweeting depends on the ability of scientists to relay the highlights of a talk or to string together multiple tweets such that they can also be read as a contiguous narrative using tools such as Storify [10]. Some simple steps to enable the wider use of live tweeting at conferences may not be widely known to scientists.

**Figure 1:**
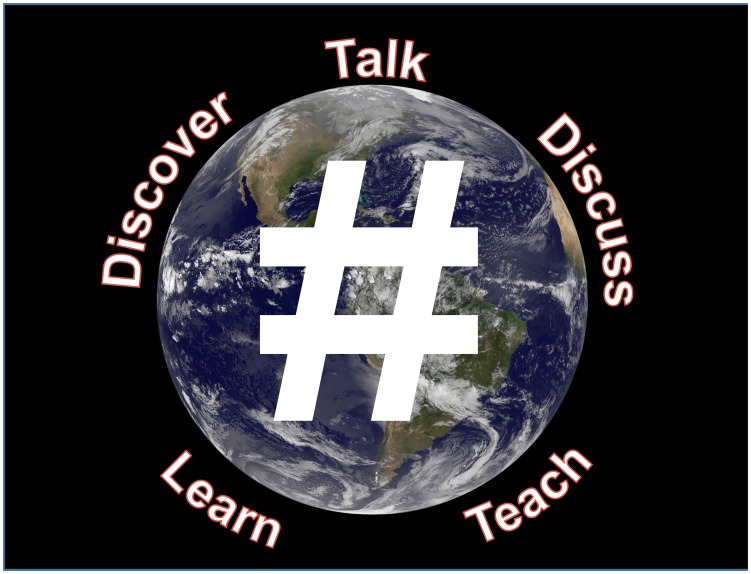
Members of an audience at a scientific conference may tweet what they hear or see, and because these messages are free and open, it has the potential to reach anyone, anywhere in the world. This has profound implications for the communication of science, enabling discovery, discussion, teaching, and learning outside of the confines of the conference itself. Image credit for globe: NASA Goddard Space Flight Center on Flickr.

For example, conferences like “Science Online” (#scioX, in which the # is a hashtag, the keyword-tagging system of Twitter that enables retrieval of all tweets about this conference) (Box 1) are at one extreme as an “unconference” [11], with multiple vibrant discussions happening during the sessions via Twitter. These discussions extend beyond the actual physical attendees, creating a parallel virtual meeting. Live tweeting is therefore a powerful tool for expanding scientific discourse to those not fortunate enough to attend a conference in person. Similarly, if a meeting has parallel sessions, tweeting then enables conference attendees or virtual conference attendees to listen in on multiple talks simultaneously. These conferences do not have to be limited to academic gatherings and may extend to those that are organized by commercial entities, which are generally more expensive to attend and very specialized. Often useful discussions happen between talks in casual environments, and tweeting those observations or conversations is probably acceptable with the agreement of both parties, unless these are private, off-the-record discussions.

**Box 1. Common Twitter Abbreviations**

# = hashtag

@ = nametag, a way to reply to someone

.@ = broadcast a tweet that begins with a nametag

RT = retweet, share something already tweeted

HT = hat tip, acknowledge or thank a source

DM = direct message

CX = correction

Tweetup = physical meeting of tweeters

Additional abbreviations can be found elsewhere: http://socialmediatoday.com/emoderation/512987/top-twitter-abbreviations-you-need-know

http://www.ogawadesign.com/services/twitter-for-your-biz/twitter-abbreviations-and-twitter-acronymns.html

http://www.webopedia.com/quick_ref/Twitter_Dictionary_Guide.asp

Acronyms for common conferences can be found here: http://www.abbreviations.com/acronyms/CONF

At the other extreme, which unfortunately is representative of most scientific conferences we have attended, there are few if any active live tweeters. This could be for several reasons: demographics of attendees, esoteric subject matter, and whether the organizer wants information to extend outside the conference halls (Gordon Conferences is one organization that may discourage tweeting on the assumption that this prevents scientists from sharing unpublished data). Sometimes the organizers of these conferences either do not actively encourage tweeting or they choose a cumbersome hashtag (Box 1) that consumes precious characters without signaling what the conference is even about (e.g., the Lysosomal Disease Network's #world_symposium, the annual conference on lysosomal storage diseases [12], which we shortened to #LDN14). Others have provided general recommendations for tweeting at academic conferences, such as rules of thumb [13], [14], dos and don'ts [15], and the types of tweets that can be useful [16]. However, we are not aware of concise efforts to describe live tweeting at science conferences other than a vaguely informative “how to tweet at conferences” [17]. An exhaustive perspective on live coverage at scientific conferences using web technologies has been described at length and focuses on bloggers in general [18], but this does not go into detail on how to use Twitter at these conferences specifically. This is important because the types of information tweeted could also be useful to followers in different spheres, such as patients, disease advocates, financial analysts, and pharmaceutical and biotech companies.

Scientists in some cases tend to be quite introverted (varying by field) so any efforts to break the ice or engage new participants at conferences are also welcome. Twitter can play an active role here to bridge or break down the gap between researcher cliques and can serve as a means to introduce you and your ideas to others in the field, without having to personally “know” them. We have found from our own experiences that Twitter interactions that initially formed online during the meeting or previous meetings can have a lasting presence in real life, forging collaborations and further expanding on discussions initiated via Twitter.

In light of those observations, it's worth proposing ten simple guidelines to encourage conference organizers, conference attendees, and anyone interested who uses Twitter to enhance the spread of scientific information beyond the physical walls of the auditoria in which meetings are held. While it is possible to add many other recommendations (such as encouraging the use of Storify to combine tweets from a meeting), we believe this is a good starting point for scientists new to Twitter and perhaps previously unwilling or unable to live tweet. While we would not claim to be the absolute authorities on Twitter use at conferences, our cumulative experiences of live tweeting have enabled us to provide a short list of recommendations. These ten simple rules are certainly ripe for future refinement or replacement as other microblogging tools are developed. Of course, it's also important to remember to enjoy the conference (if you are attending in person) and please try to add some local color to the proceedings in your tweets by describing the conference locale (using pictures if permitted). Don't be afraid to add personality while providing a voice for those not physically attending.

In the style of Twitter, we have kept these “rules or recommendations” to a maximum of 140 characters (so that they can in turn be tweeted).

## Rule 1: Short Conference Hashtag

As soon as the meeting is announced, conference organizers should claim a short (6–8 characters) descriptive # that includes the year.

## Rule 2: Promote the Hashtag

Highlight the hashtag in all conference materials online, in print, on name badges, and on Twitter if possible.

## Rule 3: Encourage Tweeting

Encourage live tweeting at the conference. Session chairs can facilitate this and relay questions from the twitterosphere.

## Rule 4: Conference Twitter Etiquette

Keep questions short and on the science, avoid grandstanding, encourage responsible tweeting, and avoid harassment or snarkiness.

## Rule 5: Conference Tweet Layout

List speaker name, affiliation and conference hashtag in the first tweet; surname or initials and meeting hashtag are sufficient thereafter.

## Rule 6: Keep Conference Discussion Flowing

Summarize presentations concisely, use hashtags for keywords, and use “@ reply” to engage individuals who can add to the discussion.

## Rule 7: Differentiate Your Opinions from the Speaker's

Separate your own comments/viewpoints on the speaker or science being described in a presentation from the speaker's own words.

## Rule 8: Bring Questions up from Outside

Check for and raise questions from those outside the conference, returning the speaker responses to positively enforce participation.

## Rule 9: Meet Other Live Tweeters Face to Face

Organize tweetups so that conference attendees can meet in person and consolidate relationships and collaborations.

## Rule 10: Emphasize Impact of Live Tweeting

Ensure that positive effects of tweeting at conferences, such as discoveries, publications, or collaborations, are highlighted.
